# *rbcL* and *matK* Earn Two Thumbs Up as the Core DNA Barcode for Ferns

**DOI:** 10.1371/journal.pone.0026597

**Published:** 2011-10-20

**Authors:** Fay-Wei Li, Li-Yaung Kuo, Carl J. Rothfels, Atsushi Ebihara, Wen-Liang Chiou, Michael D. Windham, Kathleen M. Pryer

**Affiliations:** 1 Department of Biology, Duke University, Durham, North Carolina, United States of America; 2 Institute of Ecology and Evolutionary Biology, National Taiwan University, Taipei, Taiwan; 3 Department of Botany, National Museum of Nature and Science, Tsukuba-shi, Ibaraki, Japan; 4 Division of Botanical Garden, Taiwan Forestry Research Institute, Taipei, Taiwan; Barnard College, Columbia University, United States of America

## Abstract

**Background:**

DNA barcoding will revolutionize our understanding of fern ecology, most especially because the accurate identification of the independent but cryptic gametophyte phase of the fern's life history—an endeavor previously impossible—will finally be feasible. In this study, we assess the discriminatory power of the core plant DNA barcode (*rbcL* and *matK*), as well as alternatively proposed fern barcodes (*trnH*-*psbA* and *trnL*-*F*), across all major fern lineages. We also present plastid barcode data for two genera in the hyperdiverse polypod clade—*Deparia* (Woodsiaceae) and the *Cheilanthes marginata* group (currently being segregated as a new genus of Pteridaceae)—to further evaluate the resolving power of these loci.

**Principal Findings:**

Our results clearly demonstrate the value of *matK* data, previously unavailable in ferns because of difficulties in amplification due to a major rearrangement of the plastid genome. With its high sequence variation, *matK* complements *rbcL* to provide a two-locus barcode with strong resolving power. With sequence variation comparable to *matK*, *trnL*-*F* appears to be a suitable alternative barcode region in ferns, and perhaps should be added to the core barcode region if universal primer development for *matK* fails. In contrast, *trnH*-*psbA* shows dramatically reduced sequence variation for the majority of ferns. This is likely due to the translocation of this segment of the plastid genome into the inverted repeat regions, which are known to have a highly constrained substitution rate.

**Conclusions:**

Our study provides the first endorsement of the two-locus barcode (*rbcL*+*matK*) in ferns, and favors *trnL*-*F* over *trnH*-*psbA* as a potential back-up locus. Future work should focus on gathering more fern *matK* sequence data to facilitate universal primer development.

## Introduction

In all land plants—from bryophytes to angiosperms—the typical sexual life cycle involves the alternation of a diploid sporophyte phase with a haploid gametophyte phase. Ferns and lycophytes are unique among land plants in that both sporophyte and gametophyte are not only visible to the unaided eye, but they are completely independent from one another [1]. Although diminutive and inconspicuous, fern gametophytes are key players in fern ecology and biogeography: many are thought to have wider geographic distributions than their sporophytic counterparts [2]–[4], some can exist indefinitely without producing sporophytes [5], [6], and others may be involved in hybridization events far outside the range of the parental sporophyte [7]. However, because the morphology of fern gametophytes is so reduced, it has been very difficult to confidently assign gametophytes to species, or, frequently, even to particular genera. As a result, ecological studies of gametophytes have largely been restricted to temperate regions where relatively few species exist [8], or to well-studied biological field stations in the tropics [9].

DNA barcoding offers a possible solution to this problem and could greatly improve our understanding of fern gametophytes and their biology. Recently, unknown fern gametophytes were shown to be identifiable, often to species level, by using plastid DNA sequences [5], [10], [11], suggesting that this DNA-based identification tool has the potential to be applied to large-scale ecological surveys [12]. The DNA barcoding approach has also been useful in distinguishing among fern species in the horticultural trade [13] and in Chinese herbal medicine [14], [15], two areas where species names are frequently confused. Despite these promising applications, ferns, with their critical phylogenetic position as sister to seed plants, have largely been neglected in choosing the standardized barcode for all land plants [16], [17].

Recently, the Consortium for the Barcode of Life (CBOL) approved two plastid loci, *rbcL* and *matK*, as the official DNA barcode for all land plants [18], [19], while urging further data collection on *trnH-psbA* to assess its potential to be added to the land plant barcode. CBOL's pronouncement posed a serious challenge for fern systematists and ecologists because *matK* had been recovered from only one fern species in the previous loci evaluation studies [20]–[22]. Because of the difficulties involved in obtaining *matK* data for ferns, Ebihara et al. [23] and de Groot et al. [11] proposed *trnH-psbA* and *trnL-F*, respectively, as possible substitutes.

In most plants, *matK* is nested within a *trnK* intron in the large single copy region of the plastid genome and can be amplified using primers targeting the flanking *trnK* exons [24], [25]; as these full-length *matK* sequences accumulate, further primer development for *matK* coding region should be relatively easy. In most ferns, however, the flanking *trnK* exons are absent [26]–[28], and the amplification of full-length *matK* is very difficult, thereby hindering primer development. Only recently has novel primer design helped to overcome this obstacle, with *matK* sequences now available for representatives from all fern families (*sensu* [29]) [28]. The primary aim of our study is to provide a broad overview of sequence variation across fern lineages for the core DNA barcode (*rbcL* and *matK*), as well as for the two alternatively proposed barcode regions (*trnH-psbA* and *trnL-F*). We then focus particular attention on two genera within the hyperdiverse polypod clade—*Deparia* (Woodsiaceae) and the *Cheilanthes marginata* group (a group of 17 species currently being segregated as a new genus of Pteridaceae; F.W. Li et al., unpublished). These case studies provide more detailed information regarding the resolving power of all four loci for species level identifications.

## Results

**Figure 1:**
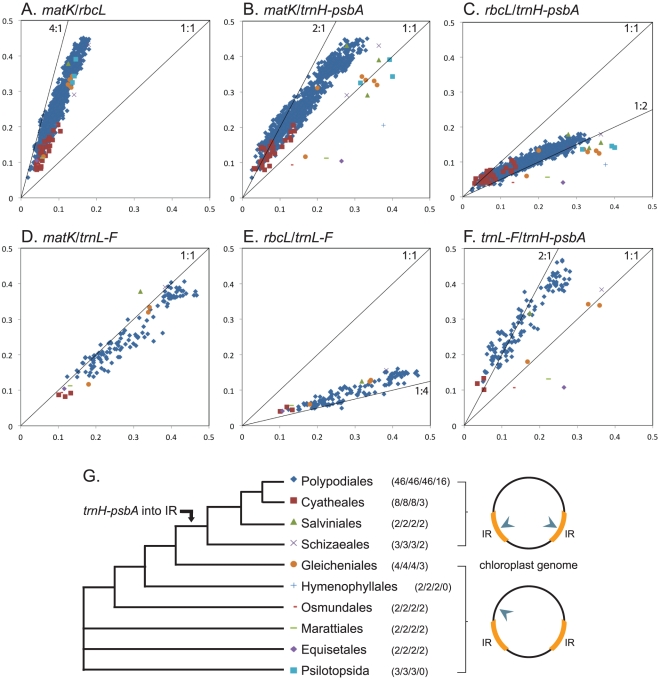
Large-scale loci comparisons across ferns. The x and y axes depict the *p*-distances calculated for each species pair within each fern order (Psilotales and Ophioglossales are combined here into class Psilotopsida). All loci comparisons are presented as y-axis vs x-axis: (A) *matK* vs *rbcL*, (B) *matK* vs *trnH-psbA*, (C) *rbcL* vs *trnH-psbA*, (D) *matK* vs *trnL-F*, (E) *rbcL* vs *trnL-F*, (F) *trnL-F* vs *trnH-psbA*. The lines in each panel (labeled with the ratios 1∶1, 1∶2, 1∶4, 4∶1 or 2∶1) are not regression lines, but are drawn to guide the eye in interpreting the results. (G) Phylogenetic relationships among fern orders, taxonomic symbols, and number of species compared per order for each locus (*matK*, *rbcL*, *trnH-psbA*, *trnL-F*, respectively). The arrow on the phylogenetic topology points to the branch where the *trnH-psbA* region of the plastid genome is predicted to have been translocated to the inverted repeat region [38]. The arrowheads point to the locations of *trnH*-*psbA* in the plastid genome.

Of the four plastid loci examined in the large-scale comparisons, *trnL-F* is the most variable across ferns, followed by *matK*, *trnH-psbA*, and then *rbcL* (Fig. 1; p<0.0001 for each comparison in Wilcoxon matched-pairs signed-rank tests, after Bonferroni correction for multiple comparisons). In contrast to its high levels of variation in most other plant lineages, *trnH-psbA* shows a markedly reduced variability in ferns (Table 1), such that 99.1% of the species pairs tested show lower divergence at *trnH-psbA* compared to *matK* (Fig. 1B), and only 5.6% are more than twice as variable as *rbcL* (Fig. 1C). This reduced variation in *trnH-psbA* is most pronounced in the recent-diverging fern lineages Cyatheales and Polypodiales (Fig. 1B–C, 1F), which together account for almost 90% of fern diversity [1].

**Table 1:** Sequence variation comparisons within different plant groups^1^.

	*Cheilanthes marginata* group (recent-diverging ferns)^2^	*Deparia* (recent-diverging ferns)^2^	Polypodiales (recent-diverging ferns)^2^	Cyatheales (recent-diverging ferns)^2^	Hymenophyllales (early-diverging ferns)^2^ [37]	Mosses^3^ [36]	*Quercus* (Fagaceae)^3^ [35]	*Alnus* (Betulaceae)^3^ [32]	*Berberis* (Berberidaceae)^3^ [31]	*Acacia* (Fabaceae)^2^ [33]	Myristicaceae^2^ [34]	Angiosperms^3^ [16]	Land plants^2^ [20]
*matK*	0.0243	0.0362	0.2667	0.1300	-	-	0.0030	0.0093	0.0050	0.0150	0.0420	0.0125	0.0113
*rbcL*	0.0099	0.0129	0.0974	0.0614	0.0358	0.0621	0.0010	0.0018	0.0010	0.0140	0.0020	0.0079	0.0129^4^
*trnH-psbA*	0.0076	0.0117	0.1632	0.0769	0.1900	0.1243	0.0103	0.0210	0.0090	0.2010	0.0600	0.0216	0.0269
*trnL-F*	-	0.0348	-	-	-	0.0973	-	-	-	-	-	-	-

This table does not intend to comprehensively cover the literature, but rather to represent a broad phylogenetic range.

1 loci variations should only be compared within each plant group; comparison among groups is not valid.

2 uncorrected *p*-distance.

3 K2P distance.

4 *rbcL*-a: a 550–600 bp subset of *rbcL.*

**Figure 2:**
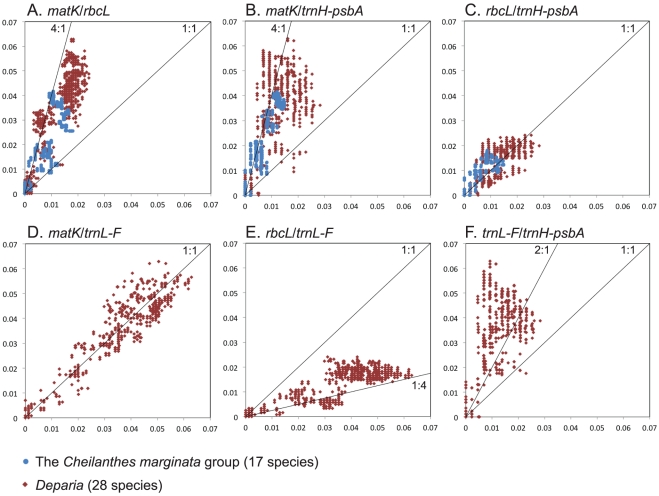
Loci comparisons within the focal polypod genera: the *Cheilanthes marginata* group and *Deparia.* The x and y axes depict the interspecific pairwise *p*-distances; the *C. marginata* group data points are represented by blue circles and *Deparia* by red diamonds. Note that *trnL-F* data were not available for the *C. marginata* group, hence they are not shown in panels D, E, F. All loci comparisons are presented as y-axis vs x-axis: (A) *matK* vs *rbcL*, (B) *matK* vs *trnH-psbA*, (C) *rbcL* vs *trnH-psbA*, (D) *matK* vs *trnL-F*, (E) *rbcL* vs *trnL-F*, (F) *trnL-F* vs *trnH-psbA*. The lines in each panel (labeled with the ratios 1∶1, 1∶4, 4∶1 or 2∶1) are not regression lines, but are drawn to guide the eye in interpreting the results.

Pairwise sequence divergence within the focal polypod genus *Deparia* is mostly comparable to the large-scale trends observed across all ferns (cf. Fig. 1A–B with Fig. 2A–B, and Fig. 1D–F with Fig. 2D–F), although *trnH-psbA* is even more conservative (cf. Fig. 1C, 1F with Fig. 2C, 2F) (Table 1). Although we do not have *trnL-F* data for the *Cheilanthes marginata* group, it shows the same general trends observed in *Deparia* for *matK*, *rbcL* and *trnH-psbA* (Fig. 2A–C). The average sequence divergence for *trnH-psbA* is lower than for all other loci tested (Table 1), and 83.2% and 58.5% of the species pairs in the *C. marginata* group and *Deparia*, respectively, exhibit a *trnH-psbA* divergence that is lower than for *rbcL* (Fig. 2C).

Finally, we examined the discriminating power for each locus and locus combination within the *C. marginata* group and within *Deparia*. Table 2 shows that among the single-locus barcodes, *matK* has the highest success rate in species discrimination and *trnH-psbA* the lowest. More importantly, when considering each locus and all combinations of loci, the highest success rate is provided by *matK*+*rbcL*, the official core DNA barcode [18], [19], [22], while including additional *trnH*-*psbA* did not increase the rate (Table 2).

**Table 2:** The power of barcoding loci and locus combinations to discriminate species.

	Percentage of species that can be uniquely discriminated (%)	
	*Cheilanthes marginata* group	*Deparia*
*matK*	47.1	75.0
*rbcL*	41.2	67.9
*trnH-psbA*	17.6	46.4
*trnL-F*	-	57.1
*matK*+*rbcL* 1	47.1	100
*matK*+*trnH-psbA*	41.2	92.9
*rbcL*+*trnH-psbA*	41.2	82.1
*matK*+*rbcL*+*trnH-psbA*	47.1	100
*matK*+*trnL-F*	-	82.1
*rbcL*+*trnL-F*	-	78.6
*matK*+*rbcL*+*trnL-F*	-	100

1 the official two-locus DNA barcode [18], [19].

## Discussion

### Toward A Consensus Barcode For Ferns

Two proposals regarding a global DNA barcode for all land plants were recently formulated and presented to CBOL [18], [19]. One consisted of *rbcL* and *matK* while the other included *rbcL*, *matK* and *trnH*-*psbA*. CBOL officially approved the *rbcL*+*matK* combination, and encouraged more data collection on *trnH*-*psbA* to assess its potential as a backup barcoding locus [18]. Because *matK* had been previously thought to be unattainable in ferns, two non-coding loci, *trnH*-*psbA* and *trnL*-*F*, were independently proposed as alternative barcoding loci [11], [23].

In this study, we provide the first thorough evaluation of the official CBOL land plant barcode (*matK* and *rbcL*) and the two alternative (*trnH*-*psbA* and *trnL*-*F*) loci for ferns. Our results build on the recent demonstration that *matK* is attainable in ferns [28], and shows that its variability is consistently high across fern lineages. Even within the species complexes represented by our two focal polypod genera, *matK* and *rbcL* together provide the highest discriminating power, supporting their use as the official core DNA barcode. It should be noted that our *matK* and *rbcL* sequences are longer than the barcode regions specified by CBOL [19]; however, we do not believe this affects our conclusions. Although universal *matK* primers remain elusive in ferns, we believe primer development will be considerably improved as more sequences become available. Therefore, it would be biased at this stage to examine and compare PCR amplification rate and sequence quality against other loci. However, if attempts to design universal *matK* primers eventually fail, our results suggest that *trnL*-*F* would be a good alternative locus since variation within *trnL-F* across ferns is comparable to that observed in *matK*.

On the other hand, we find *trnH*-*psbA* to be an unsuitable barcode for the majority of ferns, despite its obvious utility in seed plants [20], [30]–[35], mosses [36], and the early-diverging fern order, Hymenophyllales [37] (Table 1). Our results indicate that the nucleotide substitution rate for *trnH*-*psbA* is greatly reduced, especially in two recent-diverging lineages (Cyatheales and Polypodiales) that together comprise nearly 90% of fern diversity. This reduced variation in the recent-diverging ferns was also reported by Ebihara et al. [23], but it was not considered likely to be a major drawback for barcoding. However, data from our two focal polypod genera reveal that the ability of *trnH*-*psbA* to discriminate species is the lowest among the loci we tested. Considering the limited usefulness of *trnH*-*psbA* in ferns, we recommend adoption of the official CBOL land plant barcode (*rbcL*+*matK*) for future fern studies.

### An Unexpected Substitution Rate Reduction in *trnH-psbA* in Ferns

Our data provide evidence of an abrupt reduction in *trnH-psbA* sequence variation within ferns (Figure 1B–C, 1F, 2B–C, 2F; Table 1). This apparent deceleration in substitution rate seems to occur on the same branch of the fern phylogeny where the translocation of *trnH-psbA* into the inverted repeat (IR) region of the plastid genome is predicted to have occurred [38]: on the branch leading to Schizaeales, Salviniales, Cyatheales and Polypodiales (Fig. 1G, arrow). The IR region comprises two identical copies of the plastid genome that are separated by the large- and small-single copy regions (the LSR and SSR, respectively), and nucleotide substitution rates in the IR region have been shown to be dramatically slower than in either the LSR or SSR [39]–[42].

Lower substitution rates in the IR region (relative to the rest of the plastid genome) were originally attributed to the predominance of conservative rRNA genes in this region [41]; however, it has since been shown that rates are ubiquitously slow in the IR region regardless of rRNA content [42]. Two additional hypotheses have been put forth to address this rate disparity—a reduced mutation rate in the IR region, or biased gene conversion between the repeats that tend to correct mutations back to the wild-type states [42]. Invoking a reduced mutation rate may be unnecessary since it can be caused by biased gene conversion and it has been theoretically determined that a slight conversion bias could explain a reduced substitution rate [43]. An empirical study on legumes demonstrated support for this idea, showing that genes that were typically located in the IR region showed an accelerated substitution rate when the IR structure disappeared [44]. Our results from fern *trnH-psbA* provide further evidence, but from the opposite perspective—there is an apparent deceleration in substitution rate when genes are translocated into the IR region. Future statistical analyses, as well as an investigation of *ycf2*, another gene that was co-translocated with *trnH-psbA* into the IR region [38], should better characterize the dynamics of plastid genome evolution in ferns. It is nevertheless evident that because of its low substitution rate in the majority of ferns, *trnH-psbA* is not a suitable DNA barcode region for the fern lineage as a whole.

### Low Discriminating Power Within Species Complexes

Because the current DNA barcoding approach in plants relies solely on plastid loci that are mostly uniparentally inherited, it is expected that barcoding will not work well within species complexes where hybridization and polyploidy are frequent [19], [31], [45], [46]. Of our two genus case studies, the *Cheilanthes marginata* group provides a clear illustration of the problem. In addition to a series of diploid species that are easily distinguished by the official *rbcL*+*matK* barcode, the *C. marginata* group includes two species complexes, each composed of four morphological species that are polyploids of unknown origin (F.W. Li et al., unpublished) [47]. DNA barcoding only discriminates one species in the *C. angustifolia* complex, and none in the *C. marginata* complex (Supporting Information Table S1), thus recognizing less than half of the species-level biodiversity predicted on the basis of morphology. A comparable lack of discriminating power was also reported within species complexes in barcoding studies of Japanese [23] and northwestern European ferns [11].

One might argue that incorporating a nuclear locus (e.g., the internal transcribed spacer (ITS) region) could better solve the species complex problem. However, we are hesitant to recommend the use of nuclear loci in ferns (where polyploidy is frequent), because cloning is usually required, not only to obtain clear sequencing results but also to acquire all possible copies. In addition, although ITS has been shown to have high discriminating power in certain plants and animals [48], [49], these results needs to be interpreted with caution in ferns, where most of the ITS sequences reported for ferns to date [48]–[50] are nearly identical to ITS sequences reported for angiosperms (e.g., Asteraceae, Apiaceae or Fabaceae based on BLAST searches in April 2011). It should be noted that disentangling species complexes, which requires extensive genetic and chromosomal analyses, is beyond the expected goals of DNA barcoding. As shown here, the official CBOL land plant barcode allows the identification of most species derived through divergent evolution (as opposed to recent reticulate evolution), and such resolution should be sufficient for most applications [19].

## Conclusion

By incorporating both large-scale analyses and genus-level case studies, our study represents the first thorough evaluation of the official CBOL land plant barcode (*matK* and *rbcL*), as well as of two alternative barcode loci (*trnH*-*psbA* and *trnL*-*F*), for ferns. Our results provide a strong endorsement of the two-locus barcode (*rbcL*+*matK*) in ferns, and favor *trnL*-*F* over *trnH*-*psbA* as a potential back-up locus. The dramatically reduced variation observed in *trnH*-*psbA* is likely due to its translocation into the IR region of the plastid genome. Future work should focus on gathering more *matK* sequences for improved primer development, as well as examining PCR amplification and sequencing quality.

## Materials and Methods

### Sampling

To assess the discriminatory power of potential DNA barcoding loci at a broad scale, we assembled sequences of *rbcL*, *matK* and *trnH-psbA* from 74 fern species, including representatives from each of the 37 families (*sensu* [29]), and *trnL-F* sequences from 32 species representing 19 families (Supporting Information Table S2 and Table S4). More intensive inter- and intra-specific sampling was done within two focal polypod genera: *Deparia* and the *Cheilanthes marginata* group. In a recent phylogenetic analysis of the cheilanthoid ferns (M.D. Windham et al., unpublished), the *C. marginata* group was shown to be strongly monophyletic and only distantly related to the type species of *Cheilanthes* (*C. micropteris* Swartz). Taxonomic treatment of this group as a new genus is forthcoming (F.W. Li et al., unpublished). This new genus comprises 17 species, all of which were included in this study. Fifteen of the 17 species were represented by multiple individuals, giving a total sample size of 58 for the *C. marginata* group (Supporting Information Table S2). The two species that lack intra-specific sampling are known only from the type specimen or a single non-type collection (see Supporting Information Table S2) [47]. All members of the *C. marginata* group were sequenced for *matK*, *rbcL* and *trnH-psbA.* The genus *Deparia* comprises approximately 50 species currently assigned to the Woodsiaceae in the eupolypod II lineage [29], [51], [52], and 28 of these were sampled for this study. Three *Deparia* species included intra-specific sampling (42 individuals in total; Supporting Information Table S2 and Table S4), and two varieties were treated as distinct species for the species discrimination tests (Table 2). Four loci, *matK*, *rbcL*, *trnH-psbA*, and *trnL*-*F* were sequenced in *Deparia*. Voucher information for all taxa included in this study are provided in the Supporting Information Table S2 and Table S4, along with their GenBank sequence accessions.

### DNA Extraction, Amplification and Sequencing

For the *Cheilanthes marginata* group, genomic DNA was extracted using QIAGEN DNeasy Plant Mini Kits following published protocols [51]. DNA extractions in *Deparia* were done using a modified CTAB procedure [53]. All primers and PCR conditions used in this study are reported in Supporting Information Table S3. For the large-scale locus comparisons, 17 taxa were newly sequenced for *trnH-psbA* using “trnH2” and “psbAF” primers [54] or specially designed universal primers. In the *C. marginata* group, *trnH-psbA* amplification and sequencing were mostly done using “trnH2” and “psbAF” [54]; in *Deparia* newly designed universal primers were used. For *matK*, specific primers were designed separately for the two focal genera. Amplification and sequencing of *rbcL* for the *C. marginata* group used published primers [51], and for *Deparia* used “F1F” [55] and “1379R” [56]. *trnL*-*F* was amplified and sequenced using “f” [57] and “FernLr1”. For some of the older herbarium-derived samples in the *C. marginata* group, smaller overlapping fragments of *matK* and *rbcL* were amplified and sequenced using newly designed primers, and the final sequences assembled from contigs.

### Sequence Alignment And Barcoding Utility Assessment

For the large-scale comparison, we calculated species pairwise sequence divergence values within each fern order [29] to minimize alignment ambiguities: Equisetales, Marattiales, Ophioglossales+Psilotales, Osmundales, Hymenophyllales, Gleicheniales, Schizaeales, Salviniales, Cyatheales, and Polypodiales. *Psilotum nudum* was our sole representative of Psilotales, and hence was compared with Ophioglossales, which belongs to the same class (Psilotopsida). Sequences in each order were separately aligned, manually for *rbcL* and *matK* and using SATé 1.2 [58] or ClustalW [59] (followed by manual adjustments) for *trnH-psbA* and *trnL*-*F*. In SATé, MAFFT [60] was used as the “Aligner”, OPAL [61] as the “Merger”, and RAxML [62] with GTRGAMMAI as the tree estimator, and other parameters followed the default settings. When there were less than four sequences to be aligned, SATé was not applicable, and ClustalW was used instead (with the default settings). Alignments within the two focal genera were straightforward and done manually.

PAUP* v4.0a114 [63] was used to calculate pairwise sequence divergence (uncorrected *p*-distance). Substitutions in sites with gaps and/or missing data were distributed proportionally to unambiguous changes in PAUP*. For comparing sequence variation of different loci, Wilcoxon matched-pairs signed-rank tests were carried out using an online calculator (http://www.fon.hum.uva.nl/Service/Statistics/Signed_Rank_Test.html). To assess the discrimination power of each DNA region, we examined the ability of each locus and locus combination to uniquely discriminate a species from all others. The success rate of species discrimination is the percentage of species that could be distinguished among all possible species pairs. A pair of species was scored as successfully distinguished if the interspecific distance was always greater than zero and greater than the intraspecific distance. A Perl script was written to calculate the discrimination success rate from the PAUP* output (available upon request).

## Supporting Information

Table S1 List of species in the *Cheilanthes marginata* group that cannot be uniquely discriminated by the core DNA barcode.
(XLS)

Table S2 List of the taxa, samples, and GenBank accession used in this study, with separate sub-tables for the large-scale, *Cheilanthes marginata* group, and *Deparia* datasets.
(XLS)

Table S3 List of the primers and PCR conditions used in this study.
(XLS)

Table S4 List of the references cited in the three supporting tables.
(DOC)
