# Supplementary material for: rbcL and matK Earn Two Thumbs Up as the Core DNA Barcode for Ferns
Source: PLoS One. 2011 Oct 20;6(10):e26597. doi: 10.1371/journal.pone.0026597 (PMC3197659; doi:10.1371/journal.pone.0026597)
Supplement: Table S4 — List of the references cited in the three supporting tables. (DOC) [file pone.0026597.s004.doc]

1. Christenhusz MJ, Tuomisto H, Metzgar JS, Pryer KM (2008) Evolutionary relationships within the Neotropical, eusporangiate fern genus *Danaea* (Marattiaceae). Mol Phylogenet Evol 46: 34-48.

2. Des Marais D, Smith A, Britton D, Pryer KM (2003) Phylogenetic relationships and evolution of extant horsetails, *Equisetum*, based on chloroplast DNA sequence data (*rbcL* and *trnL-F*). Int J Plant Sci 164: 737-751.

3. Duffy AM, Kelchner SA, Wolf PG (2009) Conservation of selection on *matK* following an ancient loss of its flanking intron. Gene 438: 17-25.

4. Ebihara A, Ishikawa H, Matsumoto S, Lin S, Iwatsuki K, et al. (2005) Nuclear DNA, chloroplast DNA, and ploidy analysis clarified biological complexity of the *Vandenboschia radicans* complex (Hymenophyllaceae) in Japan and adjacent areas. Am J Bot 92: 1535-1547.

5. Ebihara A, Nitta JH, Ito M (2010) Molecular species identification with rich floristic sampling: DNA barcoding the pteridophyte flora of Japan. PLoS One 5: e15136.

6. Hauk WD, Parks CR, Chase MW (2003) Phylogenetic studies of Ophioglossaceae: evidence from *rbcL* and *trnL-F* plastid DNA sequences and morphology. Mol Phylogenet Evol 28: 131-151.

7. Hausner G, Olson R, Simon D, Johnson I, Sanders ER, et al. (2006) Origin and evolution of the chloroplast *trnK* (*matK*) intron: a model for evolution of group II intron RNA structures. Mol Biol Evol 23: 380-391.

8. James KE, Schneider H, Ansell SW, Evers M, Robba L, et al. (2008) Diversity arrays technology (DArT) for pan-genomic evolutionary studies of non-model organisms. PLoS One 3: e1682.

9. Karol KG, Arumuganathan K, Boore JL, Duffy AM, Everett KD, et al. (2010) Complete plastome sequences of *Equisetum arvense* and *Isoetes flaccida*: implications for phylogeny and plastid genome evolution of early land plant lineages. BMC Evol Biol 10: 321.

10. Korall P, Pryer KM, Metzgar JS, Schneider H, Conant DS (2006) Tree ferns: monophyletic groups and their relationships as revealed by four protein-coding plastid loci. Mol Phylogenet Evol 39: 830-845.

11. Korall P, Conant D, Metzgar J, Schneider H, Pryer KM (2007) A molecular phylogeny of scaly tree ferns (Cyatheaceae). Am J Bot 94: 873-886.

12. Kuo LY, Li FW, Chiou WL, Wang CN (2011) First insights into fern *matK* phylogeny. Mol Phylogenet Evol 59: 556-566.

13. Li CX, Lu SG (2006) Phylogenetic analysis of Dryopteridaceae based on chloroplast *rbcL* sequences. Acta Phytotax Sin 44: 503-515.

14. Li FW, Kuo LY, Huang YM, Chiou WL, Wang CN (2010) Tissue-direct PCR, a rapid and extraction-free method for barcoding of ferns. Mol Ecol Resour 10: 92-95.

15. Lu JM, Li DZ, Gao LM, Cheng X, Wu D (2005) Paraphyly of *Cyrtomium* (Dryopteridaceae): evidence from *rbcL* and *trnL-F* sequence data. J Plant Res 118: 129-135.

16. Madeira PT, Pemberton RW, Center TD (2008) A molecular phylogeny of the genus *Lygodium* (Schizaeaceae) with special reference to the biological control and host range testing of *Lygodium microphyllum*. Biol Control 45: 308-318.

17. Metzgar JS, Skog JE, Zimmer EA, Pryer KM (2008) The paraphyly of *Osmunda* is confirmed by phylogenetic analyses of seven plastid loci. Syst Bot 33: 31-36.

18. Nagalingum NS, Schneider H, Pryer KM (2007) Molecular phylogenetic relationships and morphological evolution in the heterosporous fern genus *Marsilea*. Syst Bot 32: 16-25.

19. Nagalingum NS, Nowak MD, Pryer KM (2008) Assessing phylogenetic relationships in extant heterosporous ferns (Salviniales), with a focus on *Pilularia* and *Salvinia*. Bot J Linn Soc 157: 673-685.

20. Perrie LR, Bayly MJ, Lehnebach CA, Brownsey PJ (2007) Molecular phylogenetic and molecular dating of the New Zealand Gleicheniaceae. Brittonia 59: 129-141.

21. Pryer KM, Smith AR, Hunt JS, Dubuisson JY (2001) *rbcL* data reveal two monophyletic groups of filmy ferns (Filicopsida: Hymenophyllaceae). Am J Bot 88: 1118-1130.

22. Pryer KM, Schuettpelz E, Wolf PG, Schneider H, Smith AR, et al. (2004) Phylogeny and evolution of ferns (monilophytes) with a focus on the early leptosporangiate divergences. Am J Bot 91: 1582-1598.

23. Rai HS, Graham SW (2010) Utility of a large, multigene plastid data set in inferring higher-order relationships in ferns and relatives (monilophytes). Am J Bot 97: 1444-1456.

24. Roper JM, Hansen SK, Wolf PG, Karol KG, Mandoli DF, et al. (2007) The complete plastid genome sequence of *Angiopteris evecta* (G. Forst.) Hoffm. (Marattiaceae). Am Fern J 97: 95-106.

25. Rothfels CJ, Larsson A, Kuo LY, Korall P, Chiou WL, et al. Overcoming deep roots, fast rates, and short internodes to resolve the ancient rapid radiation of eupolypod II ferns. Syst Biol (in press).

26. Schuettpelz E, Pryer KM (2007) Fern phylogeny inferred from 400 leptosporangiate species and three plastid genes. Taxon 56: 1037-1050.

27. Shepherd LD, Perrie LR, Parris BS, Brownsey PJ (2007) A molecular phylogeny for the New Zealand Blechnaceae ferns from analyses of chloroplast *trnL*-*trnF* DNA sequences. New Zealand J Bot 45: 67-80.

28. Smith AR, Tuomisto H, Pryer KM, Hunt JS, Wolf PG (2001) *Metaxya lanosa*, a second species in the genus and fern family Metaxyaceae. Syst Bot 26: 480-486.

29. Smith AR, Pryer KM, Schuettpelz E, Korall P, Schneider H, et al. (2006) A classification for extant ferns. Taxon 55: 705-731.

30. Su YJ, Wang T, Zheng B, Jiang Y, Chen GP, et al. (2005) Genetic differentiation of relictual populations of *Alsophila spinulosa* in southern China inferred from cpDNA *trnL-F* noncoding sequences. Mol Phylogenet Evol 34: 323-333.

31. Taberlet P, Gielly L, Pautou G, Bouvet J (1991) Universal primers for amplification of 3 noncoding regions of chloroplast DNA. Plant Mol Biol 17: 1105-1109.

32. Tate JA, Simpson BB (2003) Paraphyly of *Tarasa* (Malvaceae) and diverse origins of the polyploid species. Syst Bot 28: 723-737.

33. Tsutsumi C, Kato M (2005) Molecular phylogenetic study on Davalliaceae. Fern Gaz 17: 147-162.

34. Wakasugi T, Nishikawa A, Yamada K, Sugiura M (1998) A complete nucleotide sequence of the plastid genome from a fern, *Psilotum nudum*. Endocyt Cell Res 13(Suppl.): 147.

35. Wang M, Liu H, Lu S, Liu ZL, Chen Z (2008) Systematic position of the Woodsiaceae and Peranemaceae: evidence from chloroplast *trnL-F* region sequences. Bull Bot Res 28: 175-178.

36. Wikström N, Kenrick P, Vogel J (2002) Schizaeaceae: a phylogenetic approach. Rev Palaeobot Palynol 119: 35-50.

37. Wolf P, Rowe C, Sinclair R, Hasebe M (2003) Complete nucleotide sequence of the chloroplast genome from a leptosporangiate fern, *Adiantum capillus-veneris* L. DNA Res 10: 59-65.

38. Wolf PG, Soltis PS, Soltis DE (1994) Phylogenetic relationships of dennstaedtioid ferns: evidence from *rbcL* sequences. Mol Phylogenet Evol 3: 383-392.

39. Wolf PG, Der JP, Duffy AM, Davidson JB, Grusz AL, et al. (2011) The evolution of chloroplast genes and genomes in ferns. Plant Mol Biol: 76: 251–261.

40. Zhang G, Zhang X, Chen Z, Liu H, Yang W (2007) First insights in the phylogeny of Asian cheilanthoid ferns based on sequences of two chloroplast markers. Taxon 56: 369-378.
